# Risk factors based vessel‐specific prediction for stages of coronary artery disease using Bayesian quantile regression machine learning method: Results from the PARADIGM registry

**DOI:** 10.1002/clc.23964

**Published:** 2023-01-24

**Authors:** Hyung‐Bok Park, Jina Lee, Yongtaek Hong, So Byungchang, Wonse Kim, Byoung K. Lee, Fay Y. Lin, Martin Hadamitzky, Yong‐Jin Kim, Edoardo Conte, Daniele Andreini, Gianluca Pontone, Matthew J. Budoff, Ilan Gottlieb, Eun Ju Chun, Filippo Cademartiri, Erica Maffei, Hugo Marques, Pedro de A. Gonçalves, Jonathon A. Leipsic, Sanghoon Shin, Jung H. Choi, Renu Virmani, Habib Samady, Kavitha Chinnaiyan, Peter H. Stone, Daniel S. Berman, Jagat Narula, Leslee J. Shaw, Jeroen J. Bax, James K. Min, Woong Kook, Hyuk‐Jae Chang

**Affiliations:** ^1^ CONNECT‐AI Research Center, Yonsei University College of Medicine, Yonsei University Health System Seoul South Korea; ^2^ Department of Cardiology Catholic Kwandong University International St. Mary's Hospital Incheon South Korea; ^3^ Brain Korea 21 PLUS Project for Medical Science Yonsei University Seoul South Korea; ^4^ Department of Mathematical Sciences Seoul National University Seoul South Korea; ^5^ MetaEyes Seoul South Korea; ^6^ Department of Cardiology Gangnam Severance Hospital, Yonsei University College of Medicine Seoul South Korea; ^7^ Department of Radiology New York‐Presbyterian Hospital and Weill Cornell Medicine New York City New York USA; ^8^ Department of Radiology and Nuclear Medicine German Heart Center Munich Munich Germany; ^9^ Division of Cardiology Seoul National University College of Medicine, Cardiovascular Center, Seoul National University Hospital Seoul South Korea; ^10^ Centro Cardiologico Monzino, IRCCS Milan Italy; ^11^ Department of Medicine Lundquist Institute at Harbor UCLA Medical Center Torrance California USA; ^12^ Department of Radiology Casa de Saude São Jose Rio de Janeiro Brazil; ^13^ Seoul National University Bundang Hospital Sungnam South Korea; ^14^ Department of Radiology Fondazione Monasterio/CNR Pisa Italy; ^15^ Unit of Cardiovascular Imaging Hospital da Luz, Catolica Medical School Lisbon Portugal; ^16^ Nova Medical School Lisbon Portugal; ^17^ Department of Medicine and Radiology University of British Columbia Vancouver British Columbia Canada; ^18^ Department of Cardiology Ewha Womans University Seoul Hospital Seoul South Korea; ^19^ Department of Cardiology Pusan University Hospital Busan South Korea; ^20^ Department of Pathology CVPath Institute Gaithersburg Maryland USA; ^21^ Department of Cardiology Georgia Heart Institute, Northeast Georgia Health System Georgia USA; ^22^ Department of Cardiology William Beaumont Hospital Royal Oak Michigan USA; ^23^ Department of Cardiovascular Medicine Brigham and Women's Hospital, Harvard Medical School Boston Massachusetts USA; ^24^ Department of Imaging and Medicine Cedars Sinai Medical Center Los Angeles California USA; ^25^ Icahn School of Medicine at Mount Sinai, Mount Sinai Heart, Zena and Michael A. Wiener Cardiovascular Institute, and Marie‐Josée and Henry R. Kravis Center for Cardiovascular Health New York City New York USA; ^26^ Department of Cardiology Leiden University Medical Center Leiden The Netherlands; ^27^ Department of Cardiology Severance Cardiovascular Hospital, Yonsei University College of Medicine, Yonsei University Health System Seoul South Korea

**Keywords:** cardiovascular risk factors, coronary artery disease, machine learning

## Abstract

**Background and Hypothesis:**

The recently introduced Bayesian quantile regression (BQR) machine‐learning method enables comprehensive analyzing the relationship among complex clinical variables. We analyzed the relationship between multiple cardiovascular (CV) risk factors and different stages of coronary artery disease (CAD) using the BQR model in a vessel‐specific manner.

**Methods:**

From the data of 1,463 patients obtained from the PARADIGM (NCT02803411) registry, we analyzed the lumen diameter stenosis (DS) of the three vessels: left anterior descending (LAD), left circumflex (LCx), and right coronary artery (RCA). Two models for predicting DS and DS changes were developed. Baseline CV risk factors, symptoms, and laboratory test results were used as the inputs. The conditional 10%, 25%, 50%, 75%, and 90% quantile functions of the maximum DS and DS change of the three vessels were estimated using the BQR model.

**Results:**

The 90th percentiles of the DS of the three vessels and their maximum DS change were 41%–50% and 5.6%–7.3%, respectively. Typical anginal symptoms were associated with the highest quantile (90%) of DS in the LAD; diabetes with higher quantiles (75% and 90%) of DS in the LCx; dyslipidemia with the highest quantile (90%) of DS in the RCA; and shortness of breath showed some association with the LCx and RCA. Interestingly, High‐density lipoprotein cholesterol showed a dynamic association along DS change in the per‐patient analysis.

**Conclusions:**

This study demonstrates the clinical utility of the BQR model for evaluating the comprehensive relationship between risk factors and baseline‐grade CAD and its progression.

## 1 INTRODUCTION

Cardiovascular disease (CVD) is the primary cause of morbidity and mortality worldwide, with a global burden of 17 million deaths annually.^1^ Among them, coronary artery disease (CAD) accounts for over 50% of the total deaths and this number continues to increase.^2^ Various physiological and behavioral cardiovascular (CV) risk factors have been found to be associated with the development of CAD.^3^, ^4^, ^5^ Different symptoms can present themselves according to lesion severity or location and their interrelationships.^5^ Almost 60% of patients with stable chest pain exhibit non‐obstructive stenotic CAD with much less typical angina symptoms than obstructive CAD.^6^, ^7^ In addition, various CV risk factors are associated with symptom presentation.^8^, ^9^

Coronary atherosclerosis is a chronic and progressive process; thus, detecting subclinical atherosclerosis and intervening in its early phase has significant importance for clinical outcomes.^5^, ^9^ Therefore, comprehensive studies are needed from the early to severe stages of CAD for optimized treatments. However, to date, most previous research has focused on obstructive CAD prediction via standard regression model analysis, overlooking the importance of the early stage of CAD as most deep and shallow machine learning models investigate only the average relationship between clinical outcome and risk factors. In contrast, the Bayesian quantile regression (BQR) model, a recently introduced machine learning method, is useful for analyzing the comprehensive association between clinical variables with various stages of CAD because BQR model yields multiple quantile regression curves.^10^, ^11^, ^12^, ^13^ Particularly useful for revealing hidden independent dynamic associations of target clinical variables according to quantile stages of endpoint in a complex database such as clinical data; thus, it can be applied to specific patients for tailored therapy.

Therefore, we aimed to apply the BQR model to the association analysis between graded subclinical and clinical coronary atherosclerosis and CV risk factors to evaluate vessel‐specific dynamic interrelationships.

## 2 METHODS

### 2.1 Study design and population

We analyzed the data from Progression of AtheRosclerotic PlAque DetermIned by Computed TomoGraphic Angiography IMaging (PARADIGM, NCT02803411), a prospective, international, and multicenter observational registry designed to track coronary atherosclerosis in serially acquired coronary computed tomography angiography (CCTA).^14^ Between 2003 and 2015, 2252 consecutive patients with suspected or known CAD who underwent serial CCTA at an interscan interval of ≥2 years were enrolled. The Institutional Review Boards of all participating hospitals approved this study protocol, which was conducted according to the Declaration of Helsinki revised in 2013. The need for informed consent was waived by the Severance Hospital Institutional Review Board because the study used anonymized data (approval number 2020‐3481‐001). After the exclusion of patients with non‐interpretable scans at baseline or follow‐up CCTA (*n* = 492), documented CAD before baseline CCTA (*n* = 227), and incomplete clinical information such as CV risk factors, symptom variables, and laboratory results at baseline or follow‐up CCTA (*n* = 70), 1463 patients who underwent per‐segment‐based quantitative CCTA plaque analysis including lumen diameter stenosis (DS) were included in this study.

### 2.2 Data extraction and analysis

The baseline clinical characteristics and laboratory data were used as clinical variables, and the per‐segment‐based quantitative CCTA findings were used for a set of outcomes. We performed a vessel‐wise analysis with these data at all outcome‐level settings using the Bayesian truncated quantile regression model. For the vessel‐wise analysis, all 18 coronary segments were classified into the following three vessel groups: left anterior descending (LAD), left circumflex (LCx), and right coronary artery (RCA). The largest quantitative DS measurement in each vessel (LAD, LCx, or RCA) was regarded as the representative value for each vessel, and the largest DS among the vessels was regarded as the representative value for each patient. Most often, the LAD was included (*n* = 1264) followed by the RCA (*n* = 864) and the LCx (*n* = 718).

Figure S1 shows the histograms of DS values for the three vessels (LAD and LCx, and RCA) and each patient; the shapes of the histograms show that the data generating the distributions were not normally distributed and were truncated. Figure S2 shows the histograms of DS changes (defined as post‐DS minus pre‐DS divided by CCTA intervals) for the three vessels (the LAD, LCx, and RCA) and each patient.

We tested the following two models: the DS model (Model 1) and DS change model (Model 2). Multiple CV risk factors including the symptom variables were used to predict quantile DS values for the three vessels and each patient in Model 1 and also used to predict quantile DS changes in Model 2.

### 2.3 Quantile regression modeling

The quantile regression model for DS prediction (Model 1) was defined as follows:

(1) $$\begin{array}{l} \text{DS}\;\text{of}(\text{LAD},\text{LC}x,\text{RCA},\text{and}\;\text{per}‐\text{patient})\\ =\alpha +\sum_{i=1}^{7}\beta_{i}\cdot \mathrm{Baseline}s_{i}+\sum_{i=1}^{4}\gamma_{i}\cdot \mathrm{Symptom}\;\mathrm{Type}s_{i}\;+\sum_{i=1}^{3}\delta_{i}\cdot \mathrm{Lab}\;\mathrm{Exam}s_{i}+\epsilon_{\theta }\;, \end{array}$$

where ${\mathrm{Baselines}}_{i}$ were baseline CV risk factors including age, sex, body mass index (BMI), smoking, diabetes, hypertension, and dyslipidemia; ${\mathrm{Symptom Types}}_{i}$ were categorical risk factors denoting the types of patients’ symptoms comprised “typical angina, atypical angina, Noncardiac pain, and others” with “asymptotic” as the reference category; ${\mathrm{Lab Exams}}_{i}$ were continuous variables from laboratory examinations including high‐density lipoprotein cholesterol (HDL‐C), low‐density lipoprotein cholesterol (LDL‐C), and triglycerides (TG); $\epsilon_{\theta }$ was the error term with its θth quantile equal to zero (in our study, θ were 10%, 25%, 50%, 75%, and 90%).

Model 2 used the changes in DS values as the outcome variable, and the quantile regression model was specified as follows:

(2) $$\begin{array}{l} \text{DS}\;\text{change}\;\text{of}(\text{LAD},\text{LC}x,\text{RCA},\text{and}\;\text{per}‐\text{patient})\\ =\alpha +\sum_{i=1}^{7}\beta_{i}\cdot \mathrm{Baseline}s_{i}+\sum_{i=1}^{4}\gamma_{i}\cdot \mathrm{Symptom}\;\mathrm{Type}s_{i}\;+\sum_{i=1}^{3}\delta_{i}\cdot \mathrm{Lab}\;\mathrm{Exams}._{i}+\epsilon_{\theta }\;. \end{array}$$

### 2.4 Statistical analysis

All statistical analyses were performed using R software with package “ctqr” (version 4.1.0, R Foundation for Statistical Computing).^15^ Continuous variables were presented as means and standard deviations. Categorical variables were presented as frequencies and percentages. Prediction performance was evaluated using the area under the curve (AUC) values of the receiver operating characteristic curves.

## 3 RESULTS

### 3.1 Study population and AUC values for overall and the three major vessels

The baseline characteristics of the study population are presented in Table 1. The mean patient age was 62 years; 35.2% were women, 59.4% had hypertension, 46.3% had dyslipidemia, and 24.1% had diabetes mellitus. Most patients had atypical angina (62.2%), and typical anginal symptoms were observed in only 6.5% of the patients. AUC estimates for predicting obstructive stenosis (DS ≥ 50%) using a logistic regression model with risk factors are presented in Supplementary Figure 3. The AUC values were 0.67, 0.65, 0.78, and 0.73 for per‐patient, LAD, LCx, and RCA, respectively.

**Table 1:** Baseline patient characteristics

	Patients (*n* = 1463)
Age, years	61.8 ± 9.1
Male	1095 (64.8)
Body mass index, kg/m^2^	25.6 ± 3.4
Current smoker	320 (19.2)
Diabetes mellitus	404 (24.1)
Hypertension	993 (59.4)
Dyslipidemia	772 (46.3)
Laboratory data	
HDL cholesterol, mg/dL	49.6 ± 13.5
LDL cholesterol, mg/dL	112.5 ± 35.4
Triglycerides	145.5 ± 86.6
Symptoms	
Asymptomatic	370 (22.2)
Typical angina	109 (6.5)
Atypical angina	1038 (62.2)
Noncardiac pain	133 (8.0)
Others	139 (8.3)

*Note*: Values are presented as means ± SDs or *n* (%).

Abbreviations: HDL, high‐density lipoprotein; LDL, low‐density lipoprotein.

### 3.2 Intervessel correlation coefficients between stenosis measures

Table S1 shows the intervessel correlation coefficient estimates of the stenosis measures, revealing that the DSs of the three vessels were weakly correlated (<0.3). The low DS correlations between the vessels suggest the necessity of a per‐vessel analysis of DS for a more precise CAD diagnosis.

### 3.3 BQR analysis for DS and DS change according to CV risk factors

**Figure 1:**
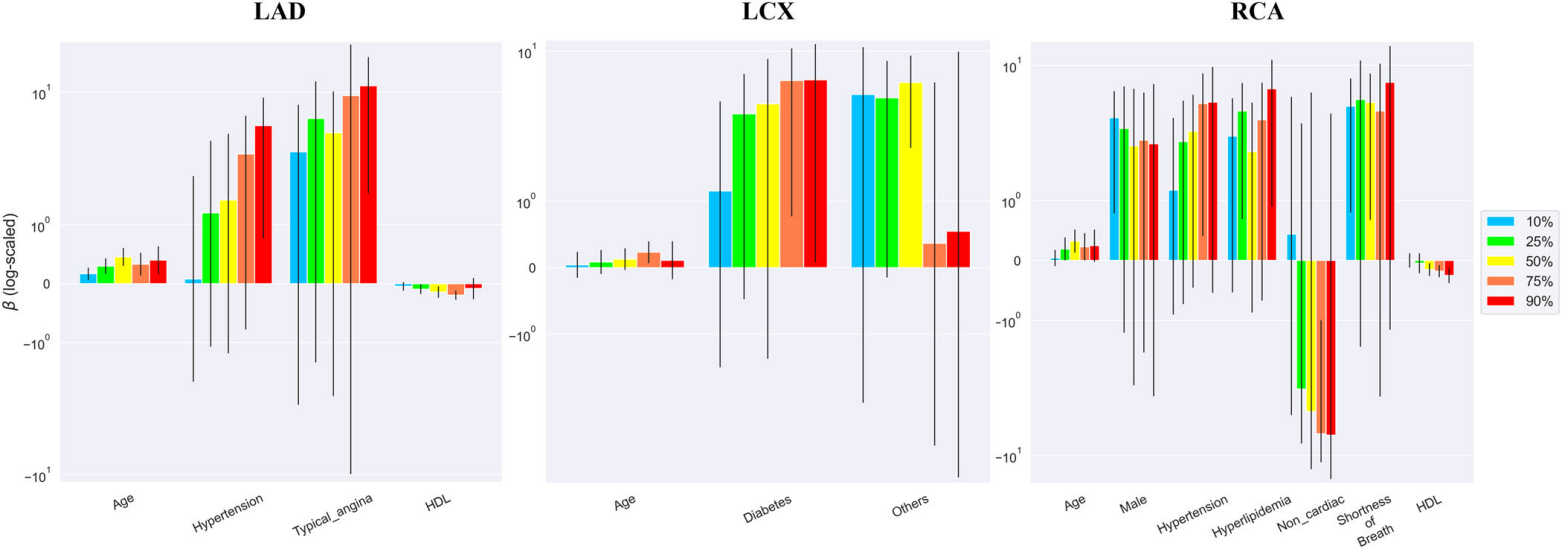
Bayesian quantile regression analysis for DS in the three vessels (LAD, LCx, and RCA). Error bar charts of the coefficient estimates with 95% confidence intervals for the selected risk factors in the three vessels are presented. Risk factors with at least one statistically significant estimate among the five quantiles (10%, 25%, 50%, 75%, and 90%) of DS were chosen using the Bayesian quantile regression model (Model 1). The y‐axis of the error bar charts is log‐scaled. DS, diameter stenosis; HDL, high‐density lipoprotein cholesterol; LAD, left anterior descending coronary artery; LCx, left circumflex coronary artery; RCA, right coronary artery.

**Figure 2:**
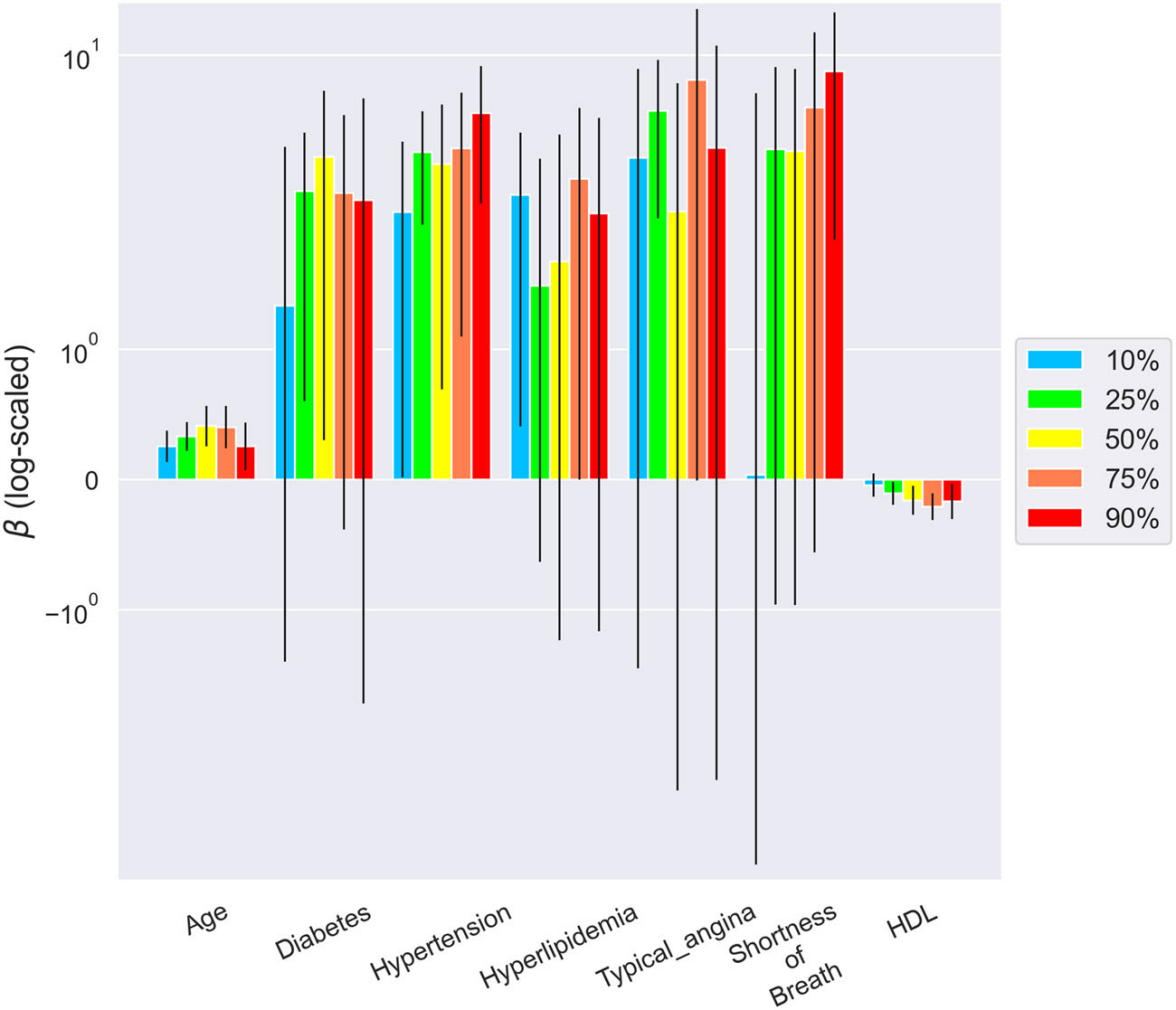
Bayesian quantile regression analysis for DS in per‐patient. The error bar chart of the coefficient estimates with 95% confidence intervals for the selected risk factors per‐patient is presented. Risk factors with at least one statistically significant estimate among the five quantiles (10%, 25%, 50%, 75%, and 90%) of DS were chosen using the Bayesian quantile regression model (Model 1). The y‐axis of the error bar chart is log‐scaled. DS, diameter stenosis; HDL, high‐density lipoprotein cholesterol.

**Figure 3:**
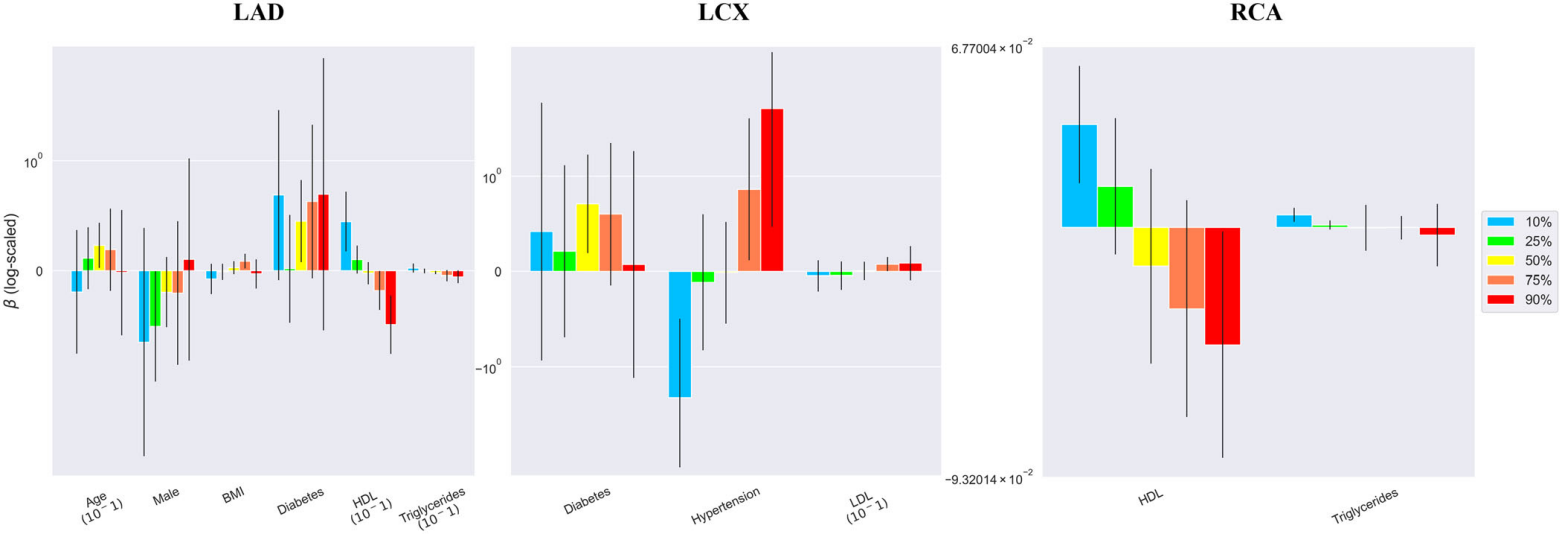
Bayesian quantile regression analysis for DS change in the three vessels (LAD, LCx, and RCA). Error bar charts of the coefficient estimates with 95% confidence intervals for the selected risk factors in the three vessels are presented. Risk factors with at least one statistically significant estimate among the five quantiles (10%, 25%, 50%, 75%, and 90%) of DS change were chosen using the Bayesian quantile regression model (Model 2). The y‐axis of the error bar charts is log‐scaled. BMI, body mass index; DS, diameter stenosis; HDL, high‐density lipoprotein cholesterol; LAD, left anterior descending coronary artery; LCx, left circumflex coronary artery; LDL‐C, low‐density lipoprotein cholesterol; RCA, right coronary artery.

**Figure 4:**
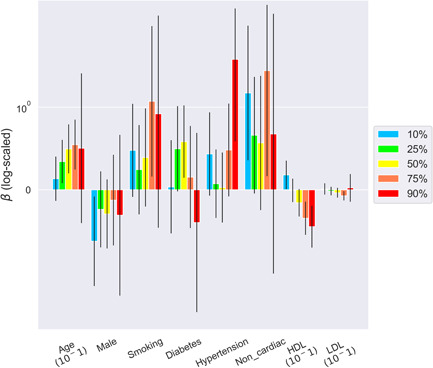
Bayesian quantile regression analysis for DS change in per‐patient. The error bar chart of the coefficient estimates with 95% confidence intervals for the selected risk factors per‐patient is presented. Risk factors with at least one statistically significant estimate among the five quantiles (10%, 25%, 50%, 75%, and 90%) of DS change were chosen using the Bayesian quantile regression model (Model 2). The y‐axis of the error bar chart is log‐scaled. DS, diameter stenosis; HDL, high‐density lipoprotein cholesterol; LDL‐C, low‐density lipoprotein cholesterol.

The quantile estimates of 10%, 25%, 50%, 75%, and 90% for the three vessels and their per‐patient values of the DS and DS changes are shown in Table S2. The mean measurements of the 90th percentiles were 41%–50% and 5.6%–7.3% in DS and DS change, respectively. Figures 1, 2, 3, 4 show the error bar charts of the coefficient estimates with 95% confidence intervals for the selected risk factors for which at least one estimate was statistically significant among the five quantiles (10%, 25%, 50%, 75%, and 90%), respectively for regression Models 1 and 2. The y‐axes were log‐scaled for clear visibility of the error bar charts.

In the per‐vessel analysis of DS, the typical anginal symptom was associated with the highest quantile (90%) of DS in the LAD; diabetes was associated with higher quantiles (75% and 90%) of DS in the LCx; dyslipidemia was associated with the highest quantile (90%) of DS in the RCA, whereas other symptoms showed some association with the LCx and RCA (Figure 1). Overall, the per‐patient analysis of DS, age, and hypertension was positively associated with all DS quantiles; in contrast, HDL‐C was negatively associated with most DS quantiles (Figure 2).

In the per‐vessel analysis of DS change, HDL‐C showed a clear and dynamic relationship, a positive association with a low level of DS change and a negative association with a high level of DS change in the LAD and RCA; hypertension also showed a dynamic relationship with DS change in the LCx and DS change severity (Figure 3). In the overall per‐patient analysis of DS change, age, smoking, and hypertension showed a tendency to increase DS change, although no consistent associations were observed. However, unlike LDL‐C, which showed no significant association with DS change, HDL‐C showed a dynamic association with DS change which changed from positive to negative with DS severity (Figure 4).

## 4 DISCUSSION

In the present study, we demonstrated the clinical utility of the Bayesian truncated quantile regression machine learning method to evaluate the comprehensive relationship between CV risk factors and baseline‐graded subclinical to clinical coronary artery stenosis and its progression.

First, while HDL‐C showed a consistent negative association with most DS levels, interestingly, the dynamic relationship was revealed for DS change severity, from positive relation to low‐level DS change and a negative association with high‐level DS change in our data set. The empirical results suggest that high HDL‐C has a preventive effect on CAD progression only for patients at a rapidly deteriorating stage. Hypertension is another CV risk factor exhibiting dynamic relation to DS change, from positive to negative, along with DS change severity. Typical angina symptoms were only associated with a high quantile of stenosis in the LAD and not in the LCx or RCA. Likewise, diabetes was strongly associated with LCx, and dyslipidemia was associated with RCA. Shortness of breath showed some relationship with a certain degree of stenosis in the LCx and RCA.

The empirical results from the BQR model provide clinical evidence supporting the implicit relationships among the risk factors. It has been known by clinical experience that LAD lesions are associated with typical anginal symptoms owing to their considerable accountability in the entire coronary perfusion^16^, ^17^; similarly, it is known by experience that LCx or RCA lesions are more likely to be associated with vague symptoms than LAD lesions.^16^, ^18^ However, to date, no scientific evidence has been provided.

In addition, HDL‐C showed a dynamic interrelationship with graded coronary stenosis and stenosis progression, which was the most distinctive utility of the BQR model that could not be achieved in any other standard regression models. Our empirical results from the BQR analysis might provide valuable clinical clues for enabling targeted management of CAD patients, especially since low HDL‐C levels could be an aggravating factor for rapid CAD progression.

Since Koenker and Bassett first introduced quantile regression models, they have been used in various research areas, such as investment, economics, and engineering, due to their multiple advantages over standard regression analysis.^19^ Quantile regression has recently been regarded as an efficient analysis tool for income and wage studies in labor economics. The Bayesian Tobit quantile regression, an advanced version of the plain quantile regression model, has been utilized to estimate outage costs in the engineering field.^10^, ^11^, ^12^

Although Wehby et al.^20^ first introduced the utility of the BQR model in the medical field by presenting the different risk factors for low and high birth weight, it is not widely adopted probably because its interpretation seems somewhat unintuitive since the concept of quantile is less familiar than means.^21^ However, with the increased interest in machine learning methods in medical research, quantile regression has recently attracted attention as a valuable data analysis tool in the medical research area.^13^ Kuhudzai et al.^22^ is the first study which indicated the impact of blood pressure risk factors in South Africa using BQR model. The study showed that the BQR model performs more accurate modeling for the hypertension estimate than classical approaches.

Although clinical models for estimating the pretest probability of CAD based on age, sex, and symptom typicality in patients with stable angina have been developed,^23^, ^24^ recent studies raised the overestimation issue of these models, potentially due to the exclusion of other important CV risk factors such as diabetes, dyslipidemia, hypertension, smoking, and obesity.^25^, ^26^ Novel imaging markers, including calcium score and multiple risk factor assessment using the machine learning method, have been evaluated to overcome this issue.^26^, ^27^ However, most studies have shown modest performance for predicting obstructive CAD and are limited to a single outcome variable of 50% DS.^25^, ^26^, ^27^

To the best of our knowledge, the present study is the first to apply BQR analysis to the prediction of CAD and especially for CAD progression, by exploring the comprehensive association between CV risk factors and various stages of CAD. This pilot study can provide a framework for the cost‐efficient utilization of previously overlooked clinical information, thereby facilitating the development of a more accurate CAD pretest probability model. Furthermore, applying BQR analysis to complex clinical data will provide a hidden pattern of certain clinical risk factors for dynamically impacting certain targeted populations with specific stages of the disease and thus will be utilized in personalized therapy.

Recent studies have shown the possibility of deep learning‐based novel methods for detecting CAD in its early stage utilizing a conventional twelve‐lead electrocardiogram (ECG).^28^ and the feasibility of convolutional neural networks for the prediction of calcium scores from traditional chest X‐ray radiography (CXR).^29^ These innovative machine learning methods and their potential combined models could turn common clinical information from ECG and CXR into vital information thereby reducing unnecessary downstream tests.

This study has several limitations. First, we only included 1463 patients with complete clinical information; most had LAD lesions and the LCx and RCA lesions were only on 465 and 340 vessels, respectively. Thus, there were insufficient data for the evaluation of the LCx or RCA. Second, although we included major CV risk factors for CAD, further specified and various CV risk factors should be included to enhance the performance of this model. Lastly, this study could not present an elaborate CAD prediction model. To develop an advanced CAD prediction analysis, balanced vessel numbers and complete clinical data are needed.

In conclusion, we introduced the BQR machine learning method in the CV field to evaluate the complex interrelationship between CV risk factors and the different stages of CAD and its progression. Using this innovative method, we comprehensively determined the dominant association of each coronary vessel with symptoms or CV risk factors, which is clinically useful.

## CONFLICT OF INTEREST

Dr Chang receives funding from by the Korea Medical Device Development Fund grant funded by the Korea government (the Ministry of Science and ICT, the Ministry of Trade, Industry and Energy, the Ministry of Health & Welfare, the Ministry of Food and Drug Safety) (Project Number: 1711139017); Dr Min receives funding from the National Institutes of Health (Grant Nos. R01 HL111141, R01 HL115150, R01 118019, and U01 HL 105907), the Qatar National Priorities Research Program (Grant No. 09‐370‐3‐089), and GE Healthcare. Dr Min served as a consultant to HeartFlow, serves on the scientific advisory board of Arineta, and has an equity interest in MDDX. Dr Bax receives unrestricted research grants from Biotronik, Medtronic, Boston Scientific, and Edwards Lifesciences. Dr Chun receives funding from National Research Foundation (NRF) grant funded by the Korea government (Ministry of Education, Science and Technology; NRF‐2015R1D1A1A01059717). Dr Leipsic is a consultant and holds stock options in HeartFlow and Circle CVI. He receives modest speaking fees from Philips and GE Healthcare. Dr Budoff receives grant support from the National Institutes of Health and GE Healthcare. Dr Marques is a Consultant and holds stock options for Cleerly Inc. Dr Samady is a cofounder and equity holder of Covanos, a consultant for Philips and Valo, and receives grant support from Phillips and St Jude Abbott/Medtronic. Dr Andreini is on the Speakers Bureau for GE Healthcare and receives grant support from GE Healthcare and Bracco. Dr Pontone receives institutional research grants from GE Healthcare, HeartFlow, Medtronic, Bracco, and Bayer. Dr Berman receives software royalties from Cedars‐Sinai. Dr Virmani has received institutional research support from 480 Biomedical, Abbott Vascular, Arterial Remodeling Technologies, BioSensors International, Biotronik, Boston Scientific, Celonova, Claret Medical, Cook Medical, Cordis, Edwards Lifesciences, Medtronic, MicroVention, OrbusNeich, ReCord, SINO Medical Technology, Spectranetics, Surmodics, Terumo Corporation, W.L. Gore and Xeltis. Dr Virmani also receives honoraria from 480 Biomedical, Abbott Vascular, Boston Scientific, Cook Medical, Lutonix, Medtronic, Terumo Corporation, and W.L. Gore, and is a consultant for 480 Biomedical, Abbott Vascular, Medtronic, and W.L. Gore. Dr Min is an employee and holds equity interest in Cleerly, Inc. He is also on the Medical Advisory Board at Arineta. The other authors report no conflicts.

## Supporting information


**Supplementary Figure 1. Histograms of DS measurements for the three vessels (LAD and LCx, and RCA) and per‐patient**. DS, diameter stenosis; LAD, left anterior descending coronary artery; LCx, left circumflex coronary artery; RCA, right coronary artery.

**Supplementary Figure 2. Histograms of DS changes for the three vessels (LAD and LCx, and RCA) and per‐patient**. Same abbreviations are used as in Supplementary Figure 1.

**Supplementary Figure 3. Logistic regression models for the three vessels (LAD, LCx, and RCA) and per‐patient for predicting obstructive stenosis (DS ≥ 50%)**. DS, diameter stenosis; LAD, left anterior descending coronary artery; LCx, left circumflex coronary artery; RCA, right coronary artery.

Supplementary information.

## Data Availability

Due to privacy and ethical concerns, neither the data nor the source of the data can be made available.
