# Correction to “Risk factors based vessel‐specific prediction for stages of coronary artery disease using Bayesian quantile regression machine learning method: Results from the PARADIGM registry”

**DOI:** 10.1002/clc.24150

**Published:** 2023-09-11

Park H‐B, Lee J, Hong Y, et al. Risk factors based vessel‐specific prediction for stages of coronary artery disease using Bayesian quantile regression machine learning method: results from the PARADIGM registry. Clin Cardiol. 2023;46:320‐327. doi:10.1002/clc.23964

The first name of the third author, Dr. Hong, was misspelled as Yongtaek in the published version. The correct spelling is Youngtaek.

We apologize for this error.

