# Supplementary figures and images for: Risk factors based vessel‐specific prediction for stages of coronary artery disease using Bayesian quantile regression machine learning method: Results from the PARADIGM registry
Source: Clin Cardiol. 2023 Jan 24;46(3):320–7. doi: 10.1002/clc.23964 (PMC10018106; doi:10.1002/clc.23964)

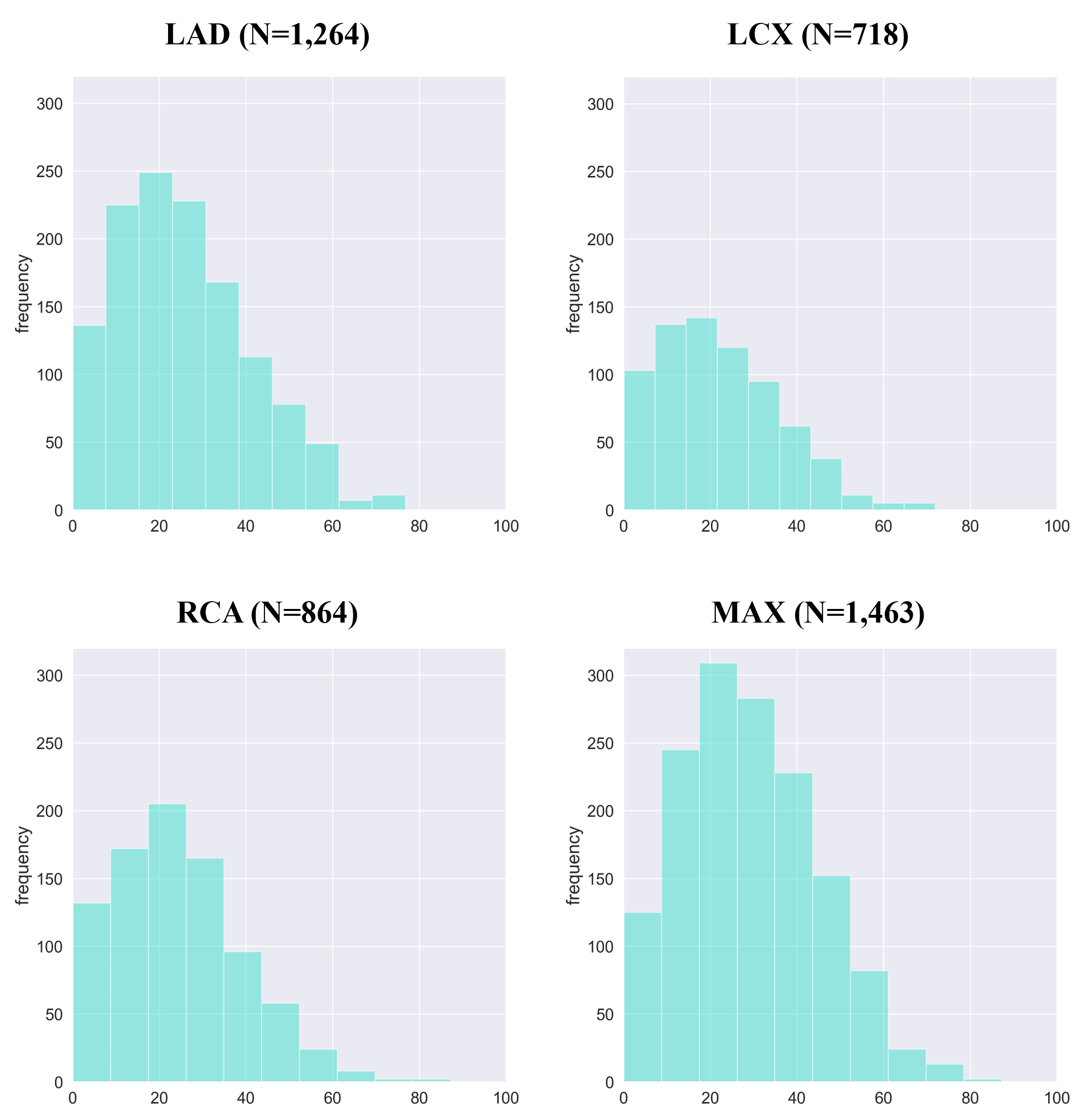

Supplement: Supplementary file 1 — Supplementary Figure 1. Histograms of DS measurements for the three vessels (LAD and LCx, and RCA) and per‐patient. DS, diameter stenosis; LAD, left anterior descending coronary artery; LCx, left circumflex coronary artery; RCA, right coronary artery. [file CLC-46-320-s004.tif]

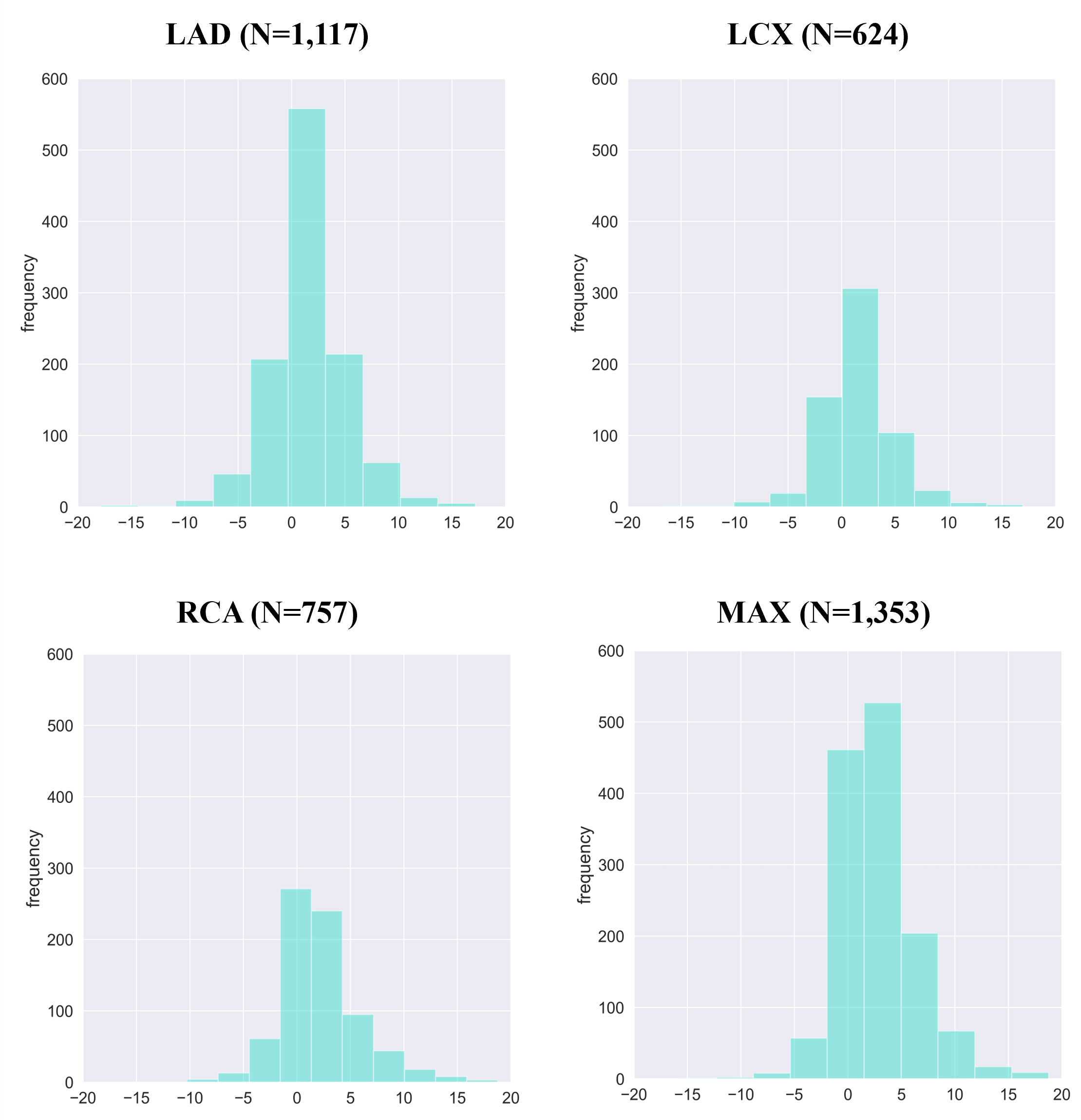

Supplement: Supplementary file 2 — Supplementary Figure 2. Histograms of DS changes for the three vessels (LAD and LCx, and RCA) and per‐patient. Same abbreviations are used as in Supplementary Figure 1. [file CLC-46-320-s002.tif]

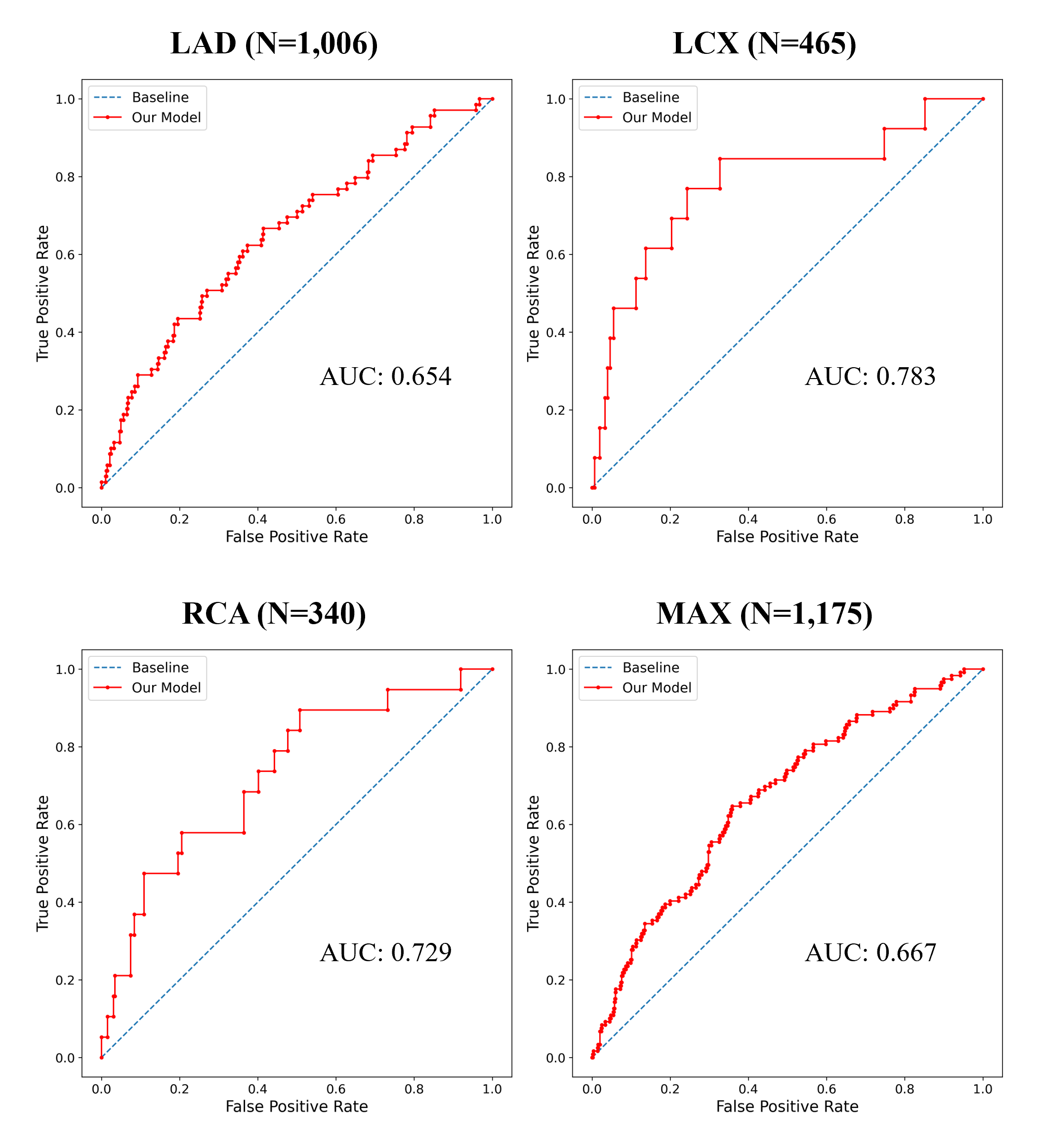

Supplement: Supplementary file 3 — Supplementary Figure 3. Logistic regression models for the three vessels (LAD, LCx, and RCA) and per‐patient for predicting obstructive stenosis (DS ≥ 50%). DS, diameter stenosis; LAD, left anterior descending coronary artery; LCx, left circumflex coronary artery; RCA, right coronary artery. [file CLC-46-320-s001.tif]
