# Supplementary material for: Risk factors based vessel‐specific prediction for stages of coronary artery disease using Bayesian quantile regression machine learning method: Results from the PARADIGM registry
Source: Clin Cardiol. 2023 Jan 24;46(3):320–7. doi: 10.1002/clc.23964 (PMC10018106; doi:10.1002/clc.23964)
Supplement: Supplementary file 4 — Supplementary information. [file CLC-46-320-s003.docx]

**Supplementary Table 1. Inter-vessel correlation coefficients of stenosis measures**

|  |  |  |  | DS |  |  |
| --- | --- | --- | --- | --- | --- | --- |
| correlation  type |  |  | (1) | (2) | (3) |  |
|  | (1) LAD |  | 1. |  |  |  |
| Pearson | (2) LCX |  | 0.27 | 1. |  |  |
|  | (3) RCA |  | 0.28 | 0.17 | 1. |  |
|  | (1) LAD |  | 1. |  |  |  |
| Kendall | (2) LCX |  | 0.17 | 1. |  |  |
|  | (3) RCA |  | 0.19 | 0.13 | 1. |  |
|  | (1) LAD |  | 1. |  |  |  |
| Spearman | (2) LCX |  | 0.26 | 1. |  |  |
|  | (3) RCA |  | 0.28 | 0.20 | 1. |  |

*DS: lumen diameter stenosis, PV: plaque volume, MPB: mean plaque burden

**Supplementary Table 2. The quantile estimates of 10%, 25%, 50%, 75%, and 90% for DS and DS change in the three vessels and per-patient**

|  | **Quantiles** | **10%** | **25%** | **50%** | **75%** | **90%** |
| --- | --- | --- | --- | --- | --- | --- |
| **DS** | **LAD** | 7.39 | 14.03 | 23.68 | 35.40 | 47.57 |
|  | **LCx** | 5.02 | 11.50 | 20.15 | 30.90 | 41.33 |
|  | **RCA** | 6.14 | 13.04 | 22.80 | 32.93 | 44.49 |
|  | **Per-patient** | 10.03 | 17.43 | 27.80 | 39.74 | 50.22 |
| **DS change** | **LAD** | −2.18 | −0.16 | 1.26 | 3.37 | 5.62 |
|  | **LCx** | −2.01 | −0.19 | 1.22 | 3.15 | 5.65 |
|  | **RCA** | −1.57 | 0.14 | 1.64 | 3.84 | 7.02 |
|  | **Per-patient** | −0.77 | 0.67 | 2.37 | 4.49 | 7.31 |

*DS, diameter stenosis; LAD, left anterior descending coronary artery; LCx, left circumflex coronary artery; RCA, right coronary artery
